# Short stature homeobox 2 methylation as a potential noninvasive biomarker in bronchial aspirates for lung cancer diagnosis

**DOI:** 10.18632/oncotarget.18056

**Published:** 2017-05-22

**Authors:** Shumin Ni, Meng Ye, Tao Huang

**Affiliations:** ^1^ The Affiliated Hospital of Ningbo University, Ningbo, Zhejiang 315020, People’s Republic of China

**Keywords:** methylation, SHOX2, lung cancer, bronchial aspirates, diagnosis

## Abstract

Gene methylation has been frequently observed in lung cancer. However, the use of methylated genes in bronchial aspirates of patients with lung cancer remains to be evaluated. The purpose of this study was to analyze whether the detection of genes with aberrant promoter methylation can be useful noninvasive biomarkers in bronchial aspirates from lung cancer. We found that the methylation status of the cyclin-dependent kinase inhibitor 2A (*P16*), Ras association domain family 1 isoform (*RASSF1A*), adenomatous polyposis coli (*APC*) and short stature homeobox 2 (*SHOX2*) genes was significantly correlated with lung cancer in bronchial aspirates. The *P16*, *RASSF1A* and *APC* methylation had a bad diagnostic effect in bronchial aspirates of patients with lung cancer compared with non-tumor controls (*P16*: sensitivity = 0.26, specificity = 0.99, area under the curve (AUC) = 0.67; *RASSF1A*: sensitivity = 0.40, specificity = 0.99, AUC = 0.66; *APC*: sensitivity = 0.17, specificity = 0.98, AUC = 0.65). The pooled sensitivity, specificity, and AUC of the *SHOX2* methylation were 0.75, 0.94, and 0.94, respectively. Moreover, when squamous cell carcinoma (SCC) was compared to adenocarcinoma (AC), the *SHOX2* gene had a significantly higher methylation rate in SCC than in AC (*P* < 0.001). Methylated *P16*, *RASSF1A*, *APC* and retinoic acid receptor beta2 (*RARβ2*) genes had similar frequencies in these two histotypes (*P* > 0.1). Our findings suggest that methylated *SHOX2* gene could be a specific and potential noninvasive biomarker using bronchial aspirates for lung cancer diagnosis, especially for SCC.

## INTRODUCTION

Lung cancer is the most common human malignant tumor and causes the highest number of cancer-related deaths worldwide. On the basis of global cancer statistics, approximately 1,824,700 new patients with lung cancer were diagnosed, leading to an estimated 1,589,900 deaths in 2012 [1]. Lung carcinoma encompasses non-small cell lung cancer (NSCLC) and small cell lung cancer (SCLC). The former accounts for approximately 85% of cases of lung cancer, mainly consisting of adenocarcinoma (AC) and squamous cell carcinoma (SCC) [2, 3].

Accumulating evidence has shown that DNA methylation, a major molecular mechanism of epigenetic changes, is correlated with human malignant tumors, including lung cancer [4–7]. Genes with aberrant DNA methylation can be noninvasive biomarkers for the detection and diagnosis of cancer [8–10]. Methylated genes were found in body fluid samples, such as plasma, urine, sera, and sputum, indicating that DNA methylation had the potential value as a biomarker in non-invasive cancer screening and diagnosis [11–13].

*P16*, consisting of an alternative reading frame of cyclin-dependent kinase inhibitor 2A (CDKN2A), is thought to function as a tumor suppressor by negatively regulating the cell cycle and inhibiting cell proliferation [14, 15]. *RASSF1A* (Ras association domain family 1 isoform) could lead to tumourigenesis associated with the regulation of the cell cycle, apoptosis, migration and adhesion [16, 17]. The adenomatous polyposis coli (*APC*) gene encodes a large multidomain protein and its dysfunction participates in tumor development [18, 19]. The human homeobox gene *SHOX2* (short stature homeobox 2) as a regulator affects skeletal, heart and nervous system development, along with embryonic morphogenesis [20, 21]. A large number of genes are shown to be frequently methylated in tissue specimens in lung cancer, including *P16*, *RASSF1A*, *APC* and *MGMT* [22, 23].

However, DNA methylation-based biomarkers using bronchial aspirates in non-invasive lung cancer detection remain to be identified. Hence, we performed the present analysis of a list of genes with aberrant DNA methylation to provide molecular clues in bronchial aspirates for the detection and diagnosis of lung cancer.

## RESULTS

### Characteristics of the included studies

**Figure 1:**
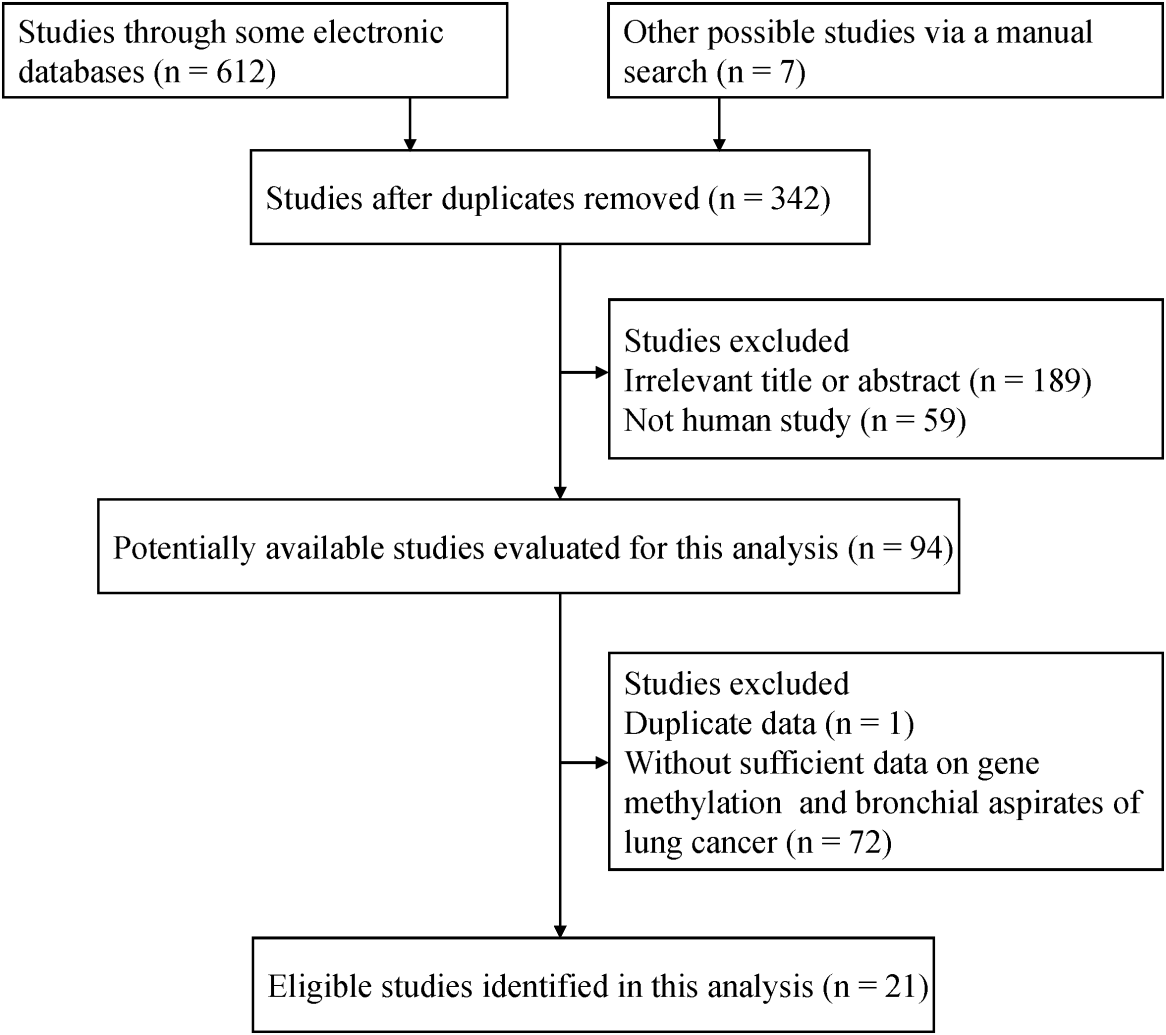
Flow diagram of the relevant literature search strategy

As depicted in Figure 1, on the basis of the inclusion criteria, final 21 eligible studies involving 20 genes were evaluated in bronchial aspirates in lung cancer [24–44]. Of these genes, four genes with more than three studies evaluated the relationship between the methylation status of the *P16*, *RASSF1A*, *APC* and *SHOX2* genes and lung cancer. The remaining 16 genes were also assessed in fewer than four studies in the current study. The general characteristics of the studies of eligibility are presented in Supplementary Table 1.

### Aberrantly methylated genes in bronchial aspirates in lung cancer

**Figure 2:**
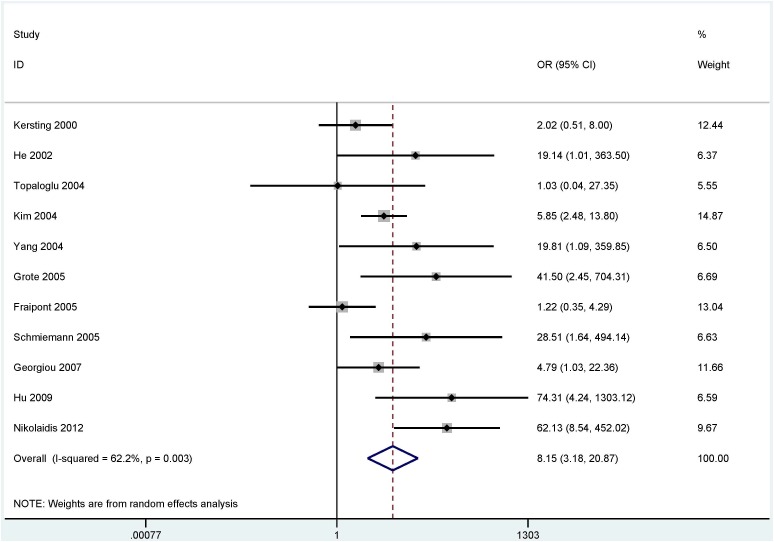
Forest plot showing the pooled OR between *P16* methylation and lung cancer in bronchial aspirates (840 lung cancer patients and 800 controls) OR = 8.15, 95% CI = 3.18 - 20.87, *P* < 0.001.

**Figure 3:**
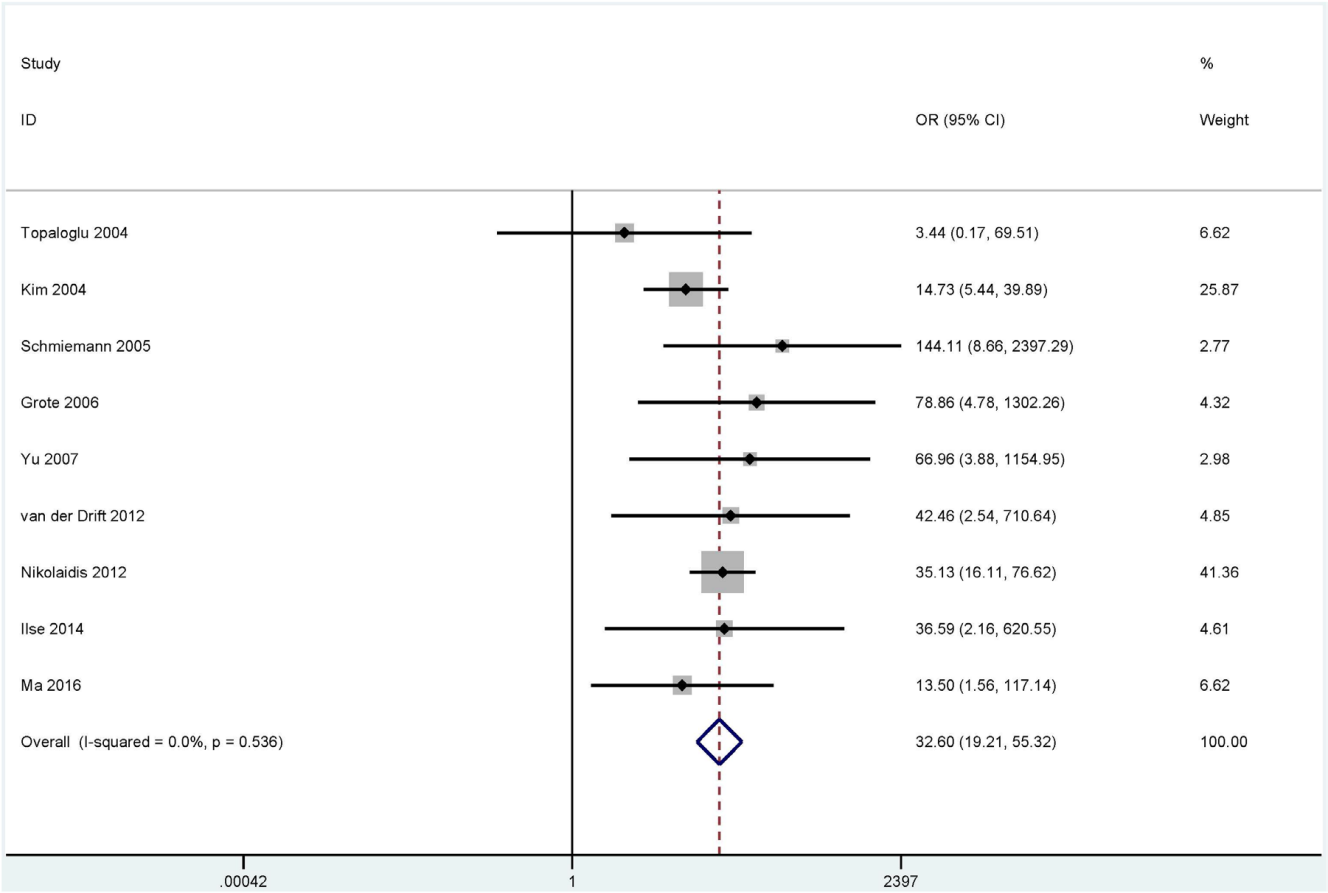
Forest plot showing the pooled OR between *RASSF1A* methylation and lung cancer in bronchial aspirates (980 lung cancer patients and 733 controls) OR = 32.60, 95% CI = 19.21 - 55.32, *P* < 0.001.

**Figure 4:**
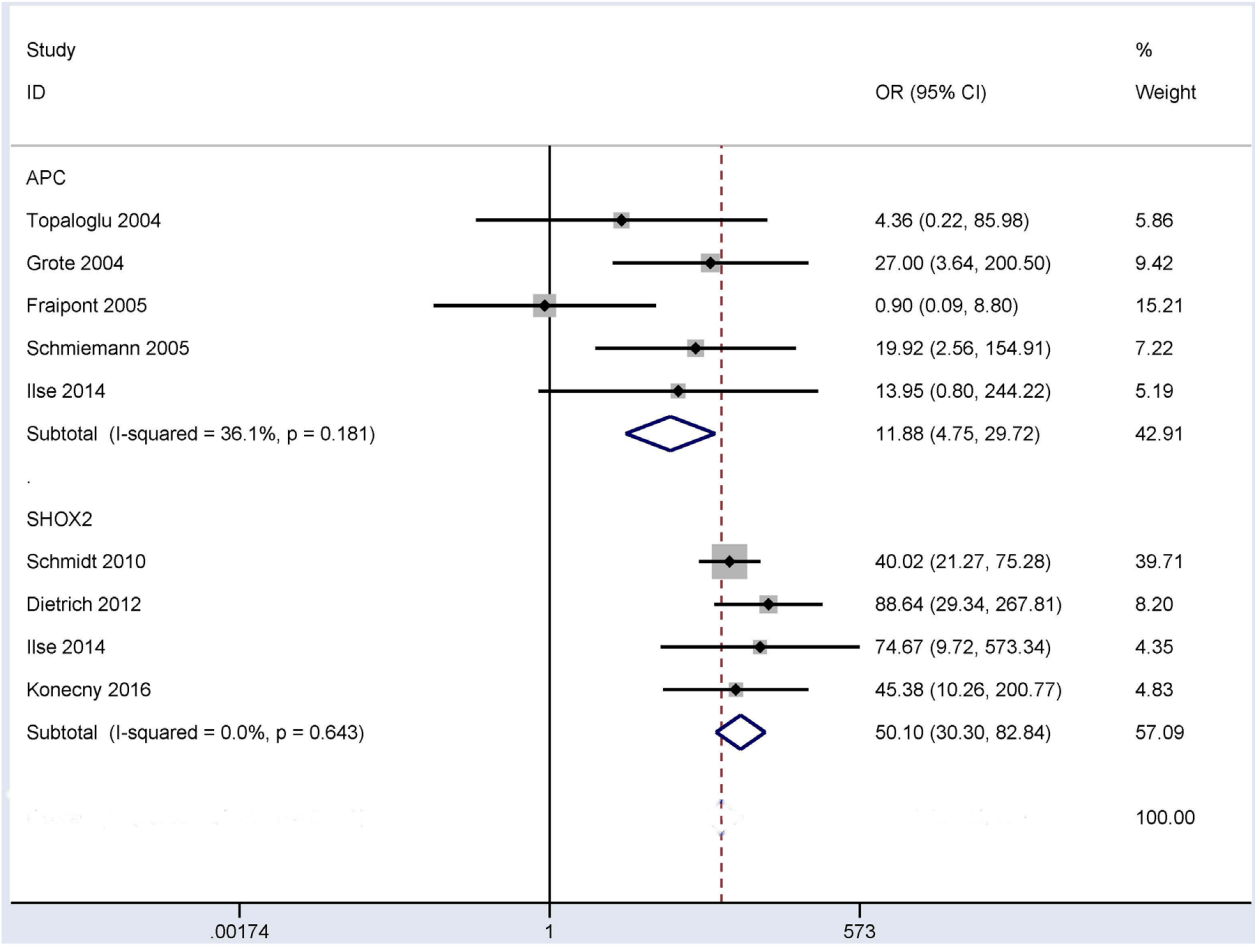
Forest plot indicating the overall OR between *APC* (360 patients with lung cancer and 273 controls) and *SHOX2* (493 patients with lung cancer and 415 controls) methylation and lung cancer in bronchial aspirates OR = 11.88, 95% CI = 4.75 - 29.72, *P* < 0.001 and OR = 50.10, 95% CI = 30.30 - 82.84, *P* < 0.001, respectively.

The results from bronchial aspirates showed that a significant correlation was observed between the methylation of the *P16* (OR = 8.15, 95% CI = 3.18 - 20.87, *P* < 0.001), *RASSF1A* (OR = 32.60, 95% CI = 19.21 - 55.32, *P* < 0.001), *APC* (OR = 11.88, 95% CI = 4.75 - 29.72, *P* < 0.001) and *SHOX2* (OR = 50.10, 95% CI = 30.30 - 82.84, *P* < 0.001) genes with lung cancer in more than four studies, including 840 lung cancer patients and 800 controls (Figure 2), 980 lung cancer patients and 733 controls (Figure 3), 360 patients with lung cancer and 273 controls (Figure 4), and 493 patients with lung cancer and 415 controls (Figure 4), respectively.

For analyses with less than four studies on the remaining 16 genes, the methylation frequency of nine genes was significantly higher in bronchial aspirates of patients with lung cancer than in controls without cancer (Supplementary Table 2).

### Subgroup analyses of the *P16* and *RASSF1A* methylation

We conducted subgroup analyses regarding the *P16* and *RASSF1A* genes to find variations in different ethnicities (Asians and Caucasians) and detection methods (methylation specific PCR (MSP) and quantitative methylation specific PCR (QMSP)) (Table 1).

**Table 1:** Subgroup analyses of methylated *P16* and *RASSF1A* genes

Gene	Subgroups	Studies	OR (95% CI)	Heterogeneity (*I^2^*; *P*)	*P* value	Cases	Controls
*P16*							
	Ethnicity						
	Caucasians	7	6.32 (1.56 - 25.53)	72.6%; 0.001	0.01	660	606
	Asians	4	11.65 (3.58 - 37.95)	25.2%; 0.260	< 0.001	180	194
	Method						
	MSP	8	4.49 (1.93 - 10.45)	45.7%; 0.075	< 0.001	347	312
	QMSP	3	46.43 (11.31 - 190.58)	0.0%; 0.902	< 0.001	493	488
*RASSF1A*							
	Ethnicity						
	Caucasians	6	40.13 (20.51 - 78.54)	0.0%; 0.593	< 0.001	810	551
	Asians	3	18.89 (8.04 - 44.39)	0.0%; 0.580	< 0.001	170	182
	Method						
	MSP	3	17.01 (6.93 - 41.78)	2.8%; 0.358	< 0.001	161	182
	QMSP	5	44.33 (22.23 - 88.39)	0.0%; 0.878	< 0.001	779	541

MSP: methylation-specific polymerase chain reaction; QMSP: quantitative methylation-specific polymerase chain reaction; OR: odds ratios; 95% CI: 95% confidence interval.

The subgroup analysis by ethnic population showed that *P16* methylation was significantly correlated with lung cancer in the Caucasian and Asian populations (OR = 6.32, *P* = 0.01 and OR = 11.65, *P* < 0.001, respectively). Based on the detection method of methylation, a significant association was found between *P16* methylation status and lung cancer in the MSP and QMSP subgroups (OR = 4.49, *P* < 0.001 and OR = 46.43, *P* < 0.001, respectively).

*RASSF1A* methylation was found to be significantly associated with lung cancer in the subgroups of the different detection methods (MSP: OR = 17.01, *P* < 0.001 and QMSP: OR = 44.33, *P* < 0.001). The subgroup analysis of ethnicity demonstrated that the methylated *RASSF1A* gene was significantly associated with lung cancer in Caucasians and Asians (OR = 40.13, *P* < 0.001 and OR = 18.89, *P* < 0.001, respectively).

### Sensitivity analysis of the *P16* methylation

We performed a sensitivity analysis by deleting an individual study to evaluate the change of the pooled OR and heterogeneity in the analysis of the *P16* methylation status with substantial heterogeneity (*I^2^* = 62.2%, *P* = 0.003). When we removed two studies by Fraipont 2005 et al., France [34] and Nikolaidis 2012 et al., and UK [27] and re-calculated the overall OR from the remaining nine studies, the result demonstrated that the combined OR of the *P16* methylation was 8.80 (95% CI = 5.08 -15.23), while heterogeneity was dramatically decreased (*I^2^* = 33.3%, *P* = 0.151).

### Aberrantly methylated genes in tumor histotypes

We further determined whether the methylation of the *P16*, *RASSF1A*, *APC*, *SHOX2* and *RARβ2* genes in the current study showed different methylation characters in SCC vs. AC and NSCLC vs. SCLC.

**Figure 5:**
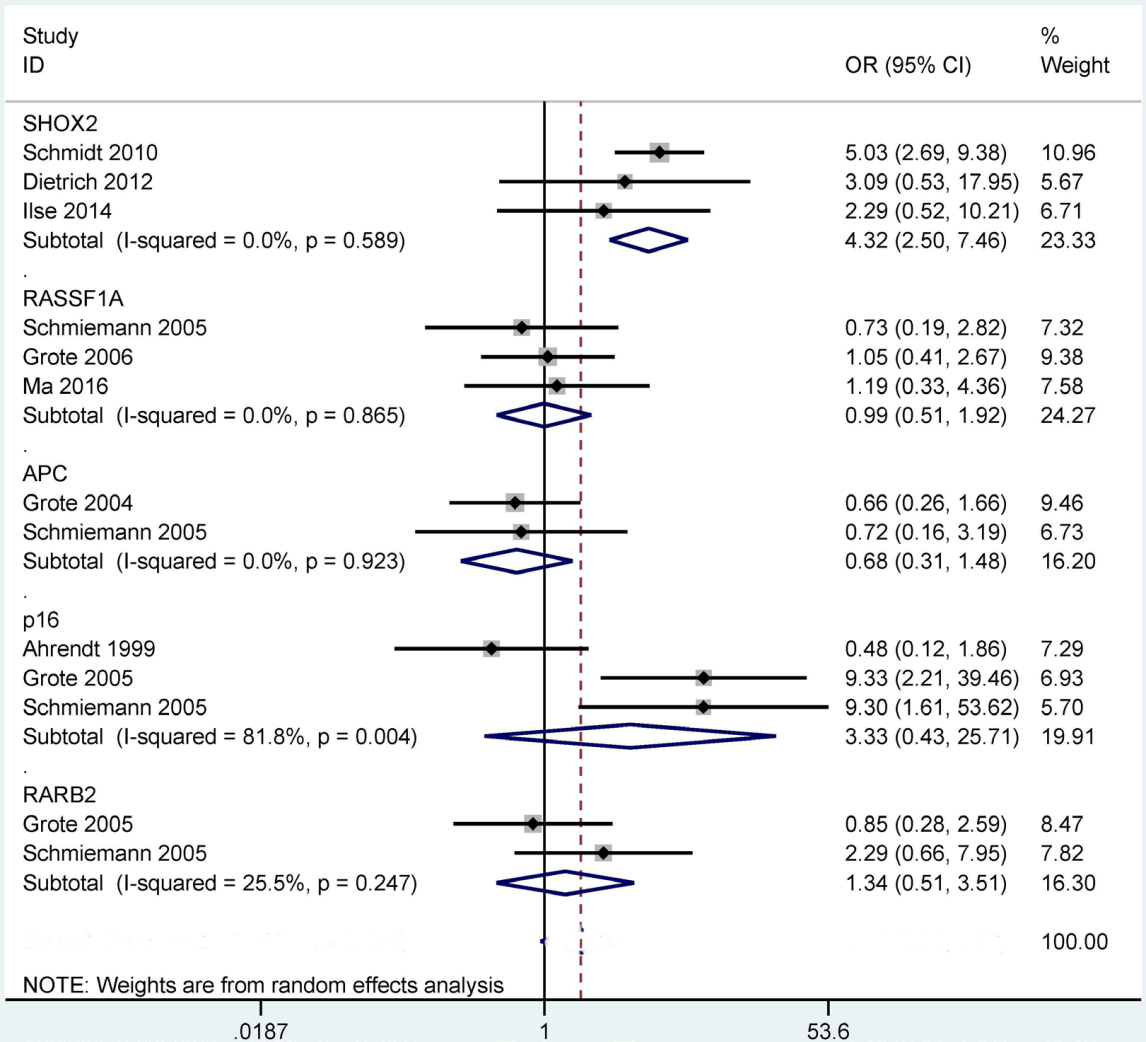
Forest plot indicating the overall OR of the methylation of the *P16*, *RASSF1A*, *APC*, *SHOX2* and *RARβ2* genes between squamous cell carcinoma (SCC) and adenocarcinoma (AC) in bronchial aspirates *SHOX2*: OR = 4.32, 95% CI = 2.50 - 7.46, P < 0.001; *P16*, *RASSF1A*, *APC* or *RARβ2* (*P* > 0.1).

When SCC was compared to AC (Figure 5), our results showed that a significant correlation was observed between the methylation of the *SHOX2* gene and tumor histology, and its methylation frequency was significantly higher in SCC than in AC (OR = 4.32, 95% CI = 2.50 - 7.46, *P* < 0.001). However, no significant relationship was found between *P16*, *RASSF1A*, *APC* or *RARβ2* methylation and these two histotypes (*P* > 0.1).

**Figure 6:**
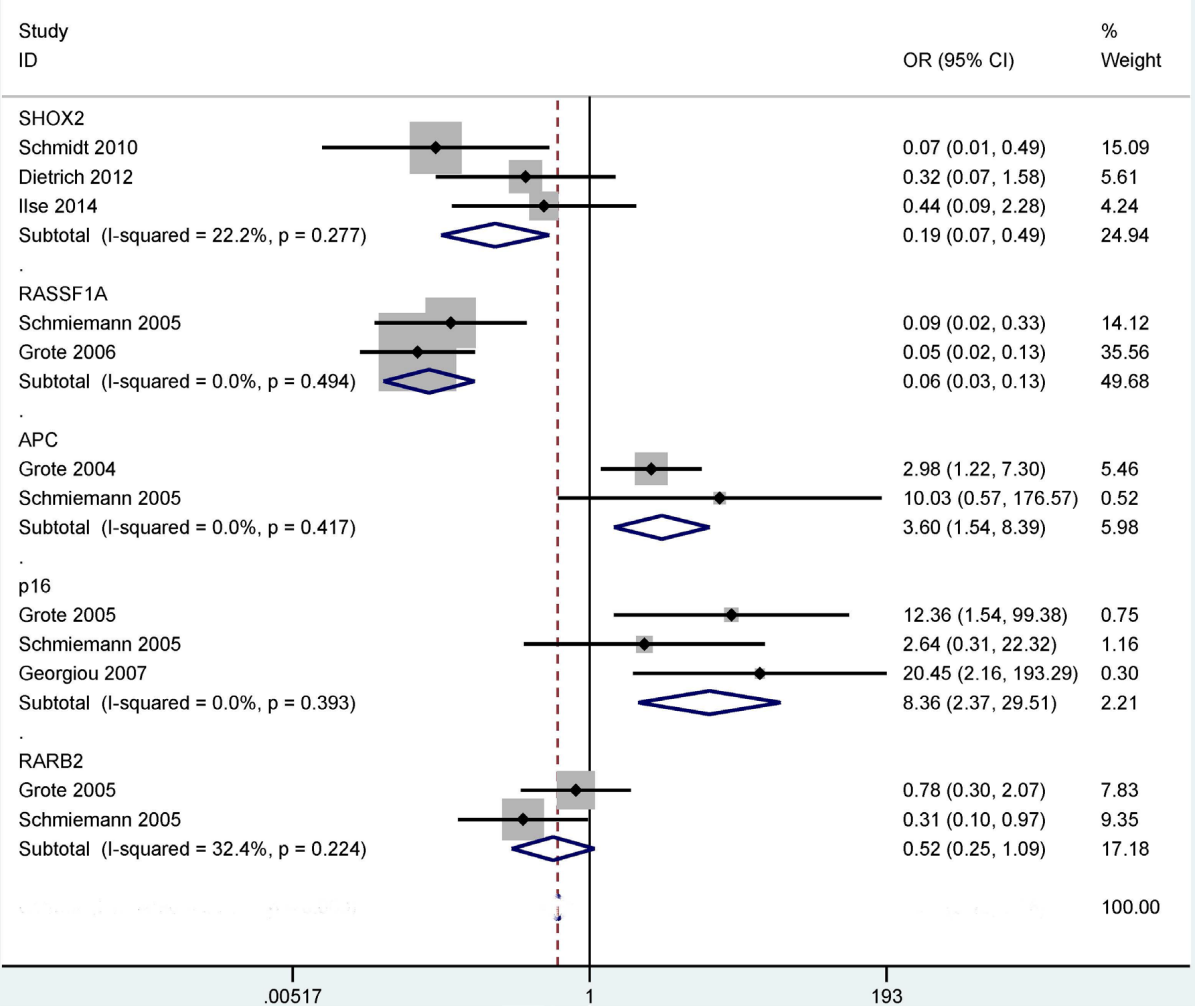
Forest plot showing the combined OR of the methylation of the *P16*, *RASSF1A*, *APC*, *SHOX2* and *RARβ2* genes between non-small cell lung cancer (NSCLC) and small cell lung cancer (SCLC) in bronchial aspirates *SHOX2*: OR = 0.19, 95% CI = 0.07 - 0.49, *P* = 0.001; *RASSF1A*: OR = 0.06, 95% CI = 0.03 - 0.13, *P* < 0.001; *P16*: OR = 8.36, 95% CI = 2.37 - 29.51, *P* = 0.001; *APC*: OR = 3.60, 95% CI = 1.54 - 8.39, *P* = 0.003; *RARβ2: P* = 0.084.

When NSCLC was compared to SCLC (Figure 6), our results demonstrated that the methylation of the *P16*, *RASSF1A*, *APC* and *SHOX2* genes was significantly correlated with tumor histology, and the methylation levels of the *SHOX2* and *RASSF1A* were significantly lower in NSCLC than in SCLC (OR = 0.19, 95% CI = 0.07 - 0.49, *P* = 0.001; and OR = 0.06, 95% CI = 0.03 - 0.13, *P* < 0.001, respectively). The methylation rate of the *P16* and *APC* genes was notably higher in NSCLC than in SCLC (OR = 8.36, 95% CI = 2.37 - 29.51, *P* = 0.001 and OR = 3.60, 95% CI = 1.54 - 8.39, *P* = 0.003, respectively). No significant association was observed between *RARβ2* methylation and tumor histology in NSCLC vs. SCLC (*P* = 0.084).

### Diagnostic effect of methylated *P16*, *RASSF1A*, *APC* and *SHOX2* genes in lung cancer

**Figure 7:**
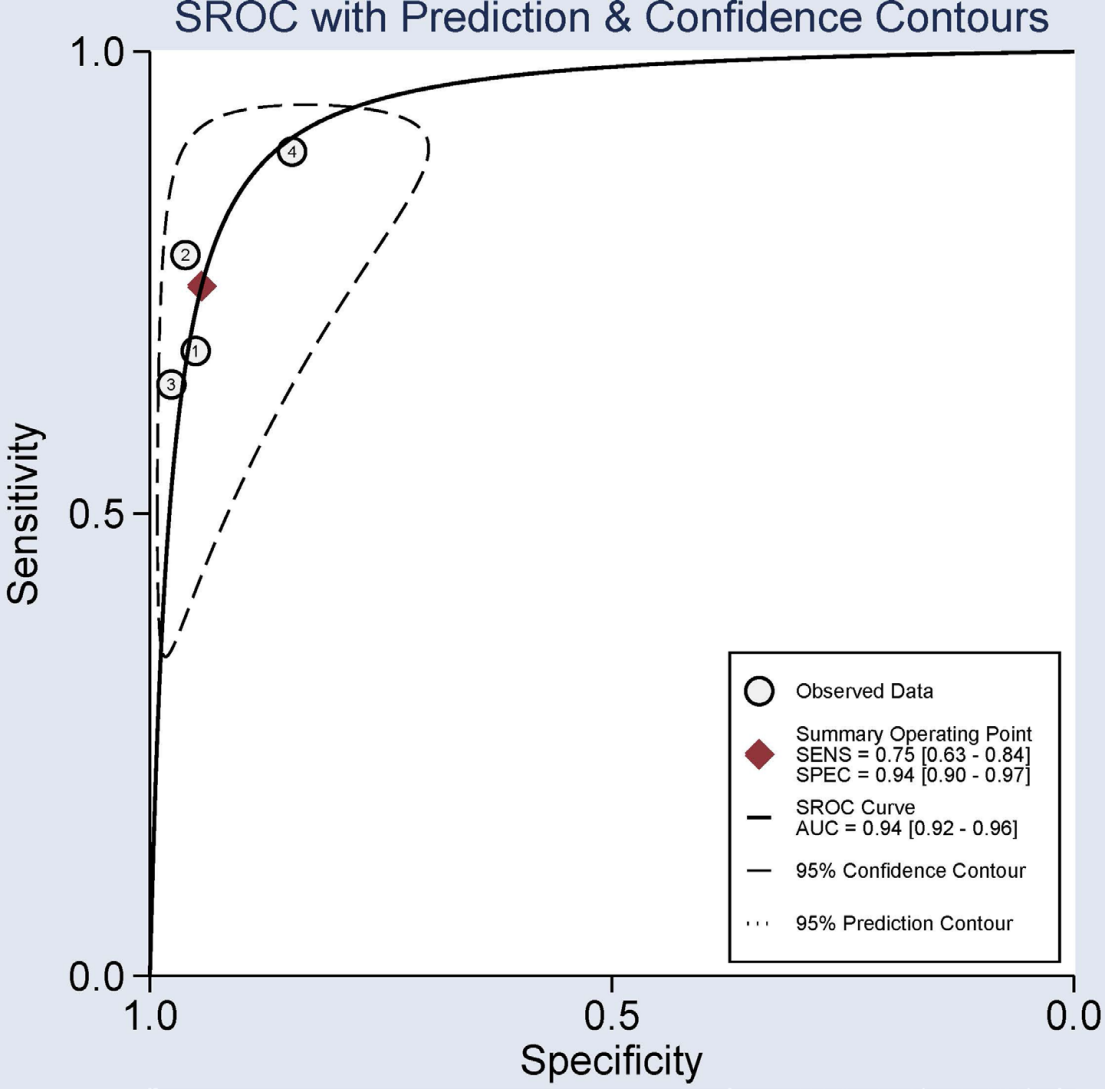
Summary receiver operating characteristics (SROC) evaluation for the potential diagnostic effect of the SHOX2 methylation using bronchial aspirates in lung cancer vs. non-tumor controls Sensitivity = 0.75 (95% CI: 0.63-0.84), specificity = 0.94 (95% CI: 0.90-0.97), and area under the curve (AUC) = 0.94 (95% CI: 0.92-0.96), suggesting that *SHOX2* methylation was a promising biomarker for lung cancer diagnosis (sensitivity = 0.75 > 0.5, specificity = 0.94 > 0.9, and AUC= 0.94 > 0.9).

This study was also conducted to further assess the diagnostic capacity of *P16*, *RASSF1A*, *APC* and *SHOX2* methylation status as non-invasive biomarkers in bronchial aspirates. When lung cancer was compared to non-tumor controls in bronchial aspirates, the methylation of the *P16* gene in bronchial aspirates from lung cancer patients vs non-tumor bronchial aspirates had a sensitivity value of 0.26 (95% CI: 0.16-0.39), a specificity value of 0.99 (95% CI: 0.92-1.00), and an AUC value of 0.67 (95% CI: 0.63-0.71) (Supplementary Figures 1–3). Methylated *RASSF1A* gene had a sensitivity value of 0.40 (95% CI: 0.34-0.46), a specificity value of 0.99 (95% CI: 0.95-1.00), and an AUC value of 0.66 (95% CI: 0.61-0.70) (Supplementary Figures 1–3). The overall sensitivity, specificity, and AUC of the *APC* methylation status were 0.17 (95% CI: 0.10-0.27), 0.98 (95% CI: 0.94-0.99), and 0.65 (95% CI: 0.61-0.69), respectively (Supplementary Figures 1–3). The pooled sensitivity, specificity, and AUC of the *SHOX2* methylation status were 0.75 (95% CI: 0.63-0.84), 0.94 (95% CI: 0.90-0.97), and 0.94 (95% CI: 0.92-0.96), respectively (Figure 7). The sensitivity value was poor for *P16*, *RASSF1A* and *APC* methylation (sensitivity < 0.5), while *SHOX2* methylation had a good sensitivity value (sensitivity = 0.75 > 0.5). The above analyses demonstrated that the methylation of the *SHOX2* gene could be a potential noninvasive biomarker based on bronchial aspirates in diagnosing lung cancer.

## DISCUSSION

Aberrant promoter methylation of tumor related genes has been reported as a promising noninvasive biomarker based on the use of feces and serum for future cancer detection and diagnosis in clinical practices [45, 46]. However, the methylated genes DNA test in samples from bronchial aspirates was not evaluated in lung cancer. A systematic study of epigenetic association publications was conducted to determine whether the use of gene methylation can become a feasible biomarker in bronchial aspirates for lung cancer screening.

Our analysis mainly focused on four tumor suppressor genes in the promoter regions (the *P16*, *RASSF1A*, *APC* and *SHOX2* genes). Reportedly, these four genes are frequently methylated in bronchial aspirates of patients with lung cancer [25, 27, 37]. However, the results with regard to the methylation levels of the *P16*, *RASSF1A*, *APC* and *SHOX2* genes in lung cancer and controls are still controversial and varied. Different methylation rates of the *P16* gene in bronchial aspirates of lung cancer patients have been shown, with a range from 3.2% [38] to 74.2% [31]. Fraipont et al. reported that the methylation frequency of the *P16* was similar in bronchial aspirates of lung cancer patients and controls without cancer [34]. The methylation levels were reported to be 28.2% in bronchial aspirates of lung cancer patients and 6.3% in controls by Kim et al. [36]. *RASSF1A* methylation frequencies ranged from 12.9% [38] to 60% [24] in bronchial aspirates of patients with lung carcinoma. Fraipont et al. reported that the *APC* gene had a low and similar methylation rate in bronchial aspirates from patients with lung cancer and controls without cancer [34]. On the other hand, *APC* had a significantly different methylation level in bronchial aspirates of patients with lung cancer and cancer-free controls (29% vs. 1.5%), as shown by Grote et al. [37]. The *SHOX2* gene had a notably higher methylation rate in bronchial aspirates of patients with lung cancer than in cancer-free controls [25, 26]. Based on eligible studies published in full text, the results showed that the methylation of the *P16*, *RASSF1A*, *APC* or *SHOX2* genes were significantly associated with lung cancer in the bronchial aspirates, suggesting that the test of methylated *P16*, *RASSF1A*, *APC* or *SHOX2* using bronchial aspirates could serve as potential noninvasive biomarkers in lung cancer.

Some studies have shown that gene methylation can be detected in body fluid samples, such as blood and feces samples, suggesting that tumor suppressor genes with aberrant DNA methylation have the potential to be noninvasive biomarkers that may contribute to the early diagnosis of cancer [45–47]. Hence, we further analyzed the diagnostic capacity of the *P16*, *RASSF1A*, *APC* and *SHOX2* methylation based on the use of bronchial aspirates in lung cancer. The results demonstrated that the *P16*, *RASSF1A* and *APC* methylation with low sensitivity (< 0.5) and AUC (< 0.8) could not distinguish lung cancer and non-tumor samples well. Interestingly, the combined sensitivity, specificity, and AUC values of the *SHOX2* methylation were 0.75, 0.94, and 0.94, respectively, which were very good (sensitivity = 0.75 > 0.5, specificity = 0.94 > 0.9, and AUC = 0.94 > 0.9), indicating that detection of the *SHOX2* methylation could be a specific noninvasive biomarker for lung cancer diagnosis using bronchial aspirates.

Although a previous study evaluated the variations between gene methylation and tumor histotypes in the comparison of SCC and AC [48], this meta-analysis did not assess the difference in the bronchial aspirates in these two histotypes. Our findings comparing SCC and AC showed that the *SHOX2* methylation was correlated with these two histotypes but not for the *P16*, *RASSF1A*, *APC* and *RARβ2* methylation. This suggested that the use of the *SHOX2* methylation as a biomarker could distinguish SCC and AC, with a significantly higher methylation level in SCC than in AC (OR = 4.32, *P* < 0.001). Moreover, in the comparison of NSCLC and SCLC, the methylation status of the *SHOX2* gene was correlated with lung cancer histology, indicating that the *SHOX2* methylation could contribute to the distinction between NSCLC and SCLC. Meanwhile, the test of the *SHOX2* methylation was notably lower in NSCLC than in SCLC (OR = 0.19, *P* = 0.001). The above analyses suggest that the detection of the *SHOX2* methylation may become a specific and promising noninvasive biomarker for the diagnosis of patients with lung cancer using bronchial aspirates, especially in SCC and SCLC.

When lung cancer patients were compared to controls, a substantial heterogeneity was measured in the *P16* gene (*I^2^* = 62.2%, *P* = 0.003). When we removed two studies [27, 34] and re-calculated the overall OR, the pooled result did not significantly change with an absence of heterogeneity, suggesting the stability of our analysis. In our study, a subgroup analysis of the testing method showed that the *P16* methylation was correlated with lung cancer in different methods. The reason of substantial heterogeneity was not very clear, but possible reasons could be inappropriate primers and the condition of detection of the *P16* methylation.

The present study had some limitations. First, we searched the abovementioned electronic databases to minimize the potential bias as completely as possible, while only available articles published in English or Chinese were included in this analysis. Publications in languages other than English and Chinese were excluded, and conference abstracts were also excluded due to insufficient data. Second, based on small sample sizes, the results regarding the comparison of the different tumor histotypes should be done to further validate the detection of the *SHOX2* gene in lung cancer histology in the future. Third, for the remaining 16 genes with small subjects, further large-scale clinical research with a large sample size is essential in bronchial aspirates. Finally, prospective large-scale studies with sufficient information, such as early and advanced stages of patients with lung cancer in bronchial aspirates, are crucial in future research.

In conclusion, our findings suggest that the *SHOX2*, *P16*, *RASSF1A* and *APC* methylation was associated with lung cancer in bronchial aspirates. The *SHOX2* methylation may become a useful noninvasive biomarker for lung cancer diagnosis, but *P16*, *RASSF1A* and *APC* methylation showed poor diagnostic effects. Moreover, only *SHOX2* methylation was significantly higher in SCC than in AC, but *P16*, *RASSF1A* or *APC* methylation had a similar level in SCC and AC. Further well-designed (multi-center) and prospective large-scale studies are required to validate the diagnostic value of gene methylation, especially for the *SHOX2* gene in bronchial aspirates of lung cancer.

## MATERIALS AND METHODS

### Search strategy

We performed a systematic literature search of the PubMed, Embase, EBSCO, Wanfang, and Cochrane Library databases until October 11th, 2016. The following key words and search terms were used: (lung cancer OR lung tumor OR lung carcinoma OR lung neoplasm) AND (methylation OR hypermethylation OR epigenetic silencing OR epigenetic inactivation) AND (bronchial* OR bronchial washings OR bronchoalveolar lavage OR bronchoalveolar lavage fluid OR BALF OR BAL). We did not apply language restrictions. To obtain other potential publications, the references of the included studies were also manually investigated in this study.

### Inclusion criteria

The selection criteria included the following: 1) patients were diagnosed with lung cancer by histopathology or endoscopy; 2) case-control or cohort design provided sufficient information regarding gene methylation in bronchial aspirates (bronchial washings, bronchoalveolar lavage fluids); and 3) the full texts of all of eligible studies were published in English or Chinese. For the presence of more than one published paper that used duplicated data, only the most recent study with more information was included in the present analysis.

### Data collection

We extracted the following information from eligible studies: the names of the first author, year of publication, country, ethnic population, histology, mean or median age, clinical stage, smoking information, gender status, methodology of the detection of gene methylation, number of case and control groups, frequency of gene methylation, and tumor histotypes. Lung cancer included NSCLC and SCLC types from the original articles in the present study.

### Data analysis

Data were calculated with Stata software (version 12.0, Stata Corporation, College Station, TX, USA). The relationships between gene methylation and lung cancer were evaluated by the combined odds ratios (ORs) and their 95% confidence interval (95% CI). The correlations between gene methylation and tumor histotypes were also determined in this study. The statistical heterogeneity was measured based on the Cochran’s Q statistic and *I*^*2*^ test [49]. A *P* value of < 0.1 for the Cochrane Q test and an *I^2^* value of > 50% were considered as substantial heterogeneity, and the random-effects model was applied for this meta-analysis; otherwise, a fixed-effects model was used [50, 51]. To evaluate the influence of an individual study on the pooled results, the omission of one study was performed by sensitivity analysis when significant heterogeneity was tested in genes with more than five studies [52]. Subgroup analyses were carried out to find the differences among the different subgroups. Additionally, according to the bivariate analysis, the pooled sensitivity, specificity, and the summary receiver operator characteristic (SROC) curve (AUC) values were utilized to further evaluate and explore the diagnostic effect of *P16*, *RASSF1A*, *APC* and *SHOX2* methylation in bronchial aspirates of patients with lung cancer. An AUC value was commonly applied to determine the accuracy of the diagnostic test in the meta-analysis [53, 54].

## SUPPLEMENTARY MATERIALS FIGURES AND TABLES




