# Supplementary material for: Short stature homeobox 2 methylation as a potential noninvasive biomarker in bronchial aspirates for lung cancer diagnosis
Source: Oncotarget. 2017 May 22;8(37):61253–63. doi: 10.18632/oncotarget.18056 (PMC5617421; doi:10.18632/oncotarget.18056)
Supplement: Supplementary file 1 [file oncotarget-08-61253-s001.pdf]

Short stature homeobox 2 methylation as a potential noninvasive biomarker in bronchial aspirates for lung cancer diagnosis

SUPPLEMENTARY MATERIALS

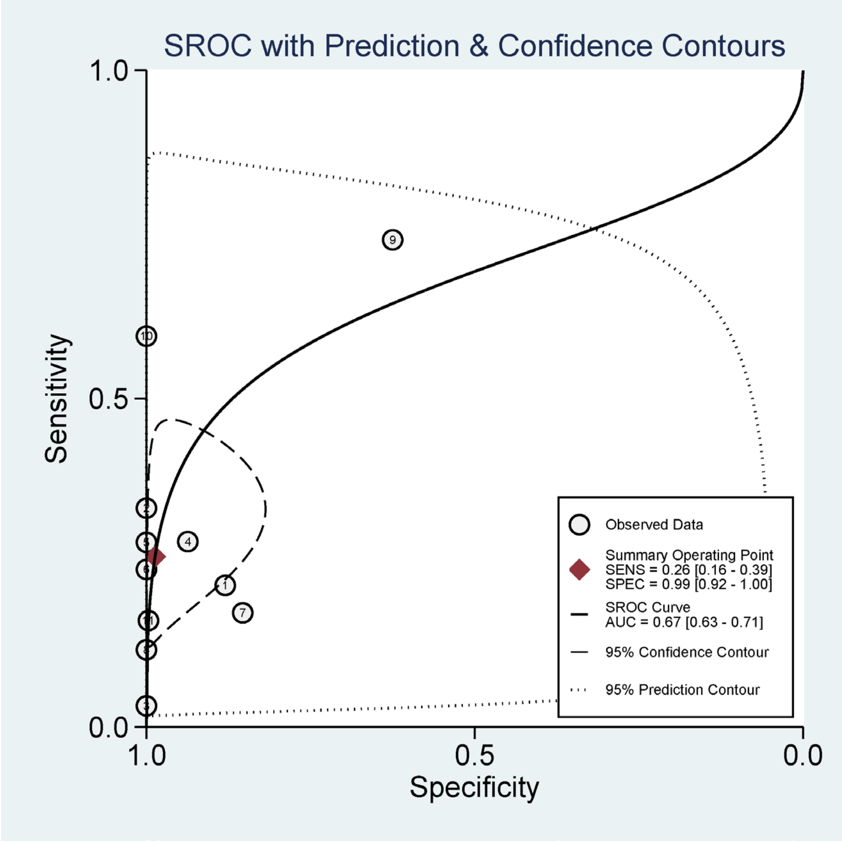

Supplementary Figure 1: Summary receiver operating characteristics (SROC) estimation for the potential diagnostic effect of the P16 methylation using bronchial aspirates in lung cancer vs. non-tumor controls.

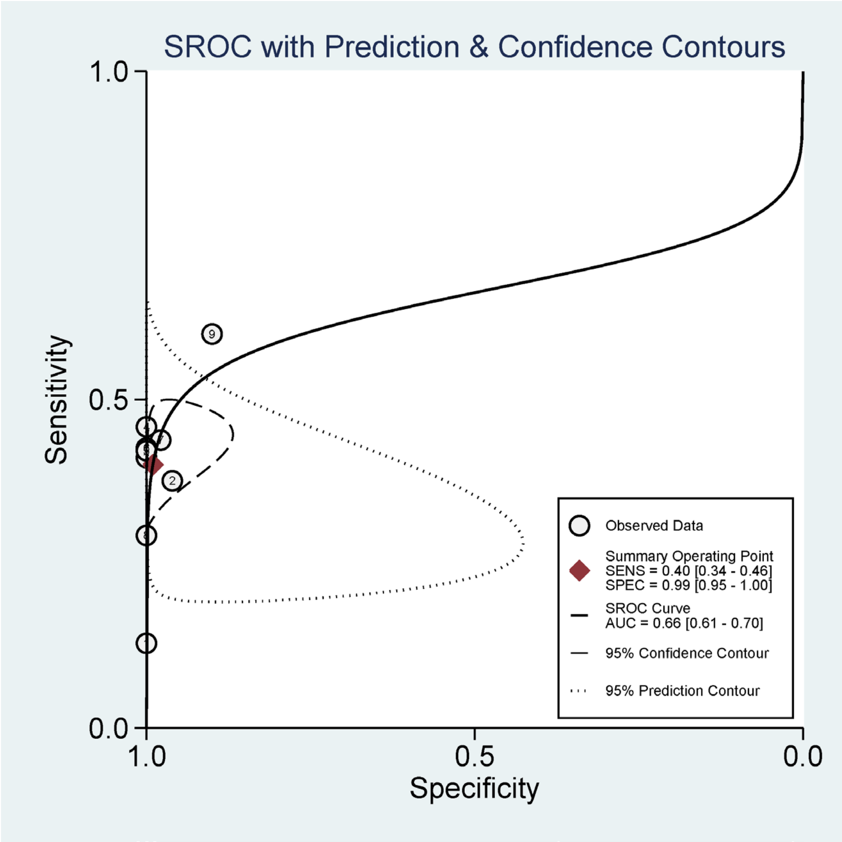

Supplementary Figure 2: Summary receiver operating characteristics (SROC) estimation for the potential diagnostic effect of the RASSF1A methylation using bronchial aspirates in lung cancer vs. non-tumor controls.

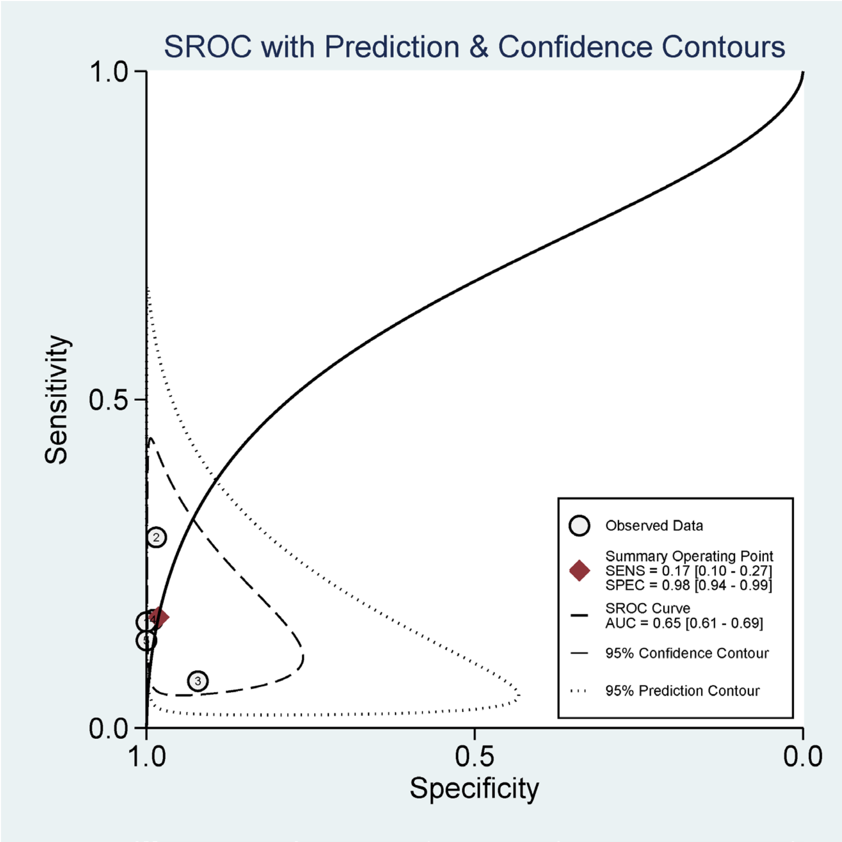

Supplementary Figure 3: Summary receiver operating characteristics (SROC) estimation for the potential diagnostic effect of the APC methylation using bronchial aspirates in lung cancer vs. non-tumor controls.

Supplementary Table 1: General characteristics of the studies of the eligibility

See Supplementary File 1

Supplementary Table 2: The summary of the association in bronchial aspirates of patients with lung cancer vs. non-tumor controls

Gene	Studies	OR (95% CI)	Heterogeneity (I^2; *P*)	*P* value	Cancer	Controls
P16	11	8.15 (3.18 - 20.87)	62.2%; 0.003	< 0.001	840	800
RASSF1A	9	32.60 (19.21 - 55.32)	0.0%; 0.536	< 0.001	980	733
APC	5	11.88 (4.75 - 29.72)	36.1%; 0.181	< 0.001	360	273
SHOX2	4	50.10 (30.30 - 82.84)	0.0%; 0.525	< 0.001	493	415
RARB2	3	5.39 (2.22 - 13.08)	63.2%; 0.099	< 0.001	189	176
FHIT	2	1.23 (0.73 - 2.05)	0.0%; 0.931	0.439	107	201
RARB	2	4.13 (2.83 - 6.04)	0.0%; 0.903	< 0.001	418	449
MGMT	2	5.84 (1.14 - 29.95)	0.0%; 0.920	0.034	55	88
CDH13	2	3.27 (0.15 - 71.57)	96.2%; < 0.001	0.452	279	340
DAPK	2	1.52 (0.69 - 3.32)	3.3%; 0.309	0.299	218	290
GSTP1	1	1.03 (0.04 - 27.35)	NA; NA	0.985	31	10
CDH1	1	15.32 (0.82 - 284.84)	NA; NA	0.067	31	10
SEMA3B	1	0.64 (0.13 - 3.17)	NA; NA	0.582	75	25
TERT	1	12.34 (8.21 - 18.54)	NA; NA	< 0.001	333	322
WT1	1	12.71 (7.54 - 21.44)	NA; NA	< 0.001	333	322
CYGB	1	3.46 (1.93 - 6.21)	NA; NA	< 0.001	333	322
p73	1	2.11 (1.12 - 3.96)	NA; NA	0.02	194	213
TMEFF	1	1.11 (0.51 - 2.38)	NA; NA	0.798	194	213
HOXA9	1	47.88 (2.60 - 882.37)	NA; NA	0.009	40	10
PCDHGB6	1	12.18 (1.41 - 105.48)	NA; NA	0.023	40	10

OR: odds ratios; 95% CI: 95% confidence interval.
