# Supplementary material for: Short stature homeobox 2 methylation as a potential noninvasive biomarker in bronchial aspirates for lung cancer diagnosis
Source: Oncotarget. 2017 May 22;8(37):61253–63. doi: 10.18632/oncotarget.18056 (PMC5617421; doi:10.18632/oncotarget.18056)
Supplement: Supplementary file 2 [file oncotarget-08-61253-s002.docx]

**Supplementary Table 1: General characteristics of the studies of the eligibility**

| Gene | First author | Country | Ethnicity | Age | Method | Smoking | Male | Stage | Cancer | Controls | SCC | AC | NSCLC | SCLC |
| --- | --- | --- | --- | --- | --- | --- | --- | --- | --- | --- | --- | --- | --- | --- |
|  |  |  |  |  |  | N | N |  | N (M %) | N (M %) | M/N | M/N | M/N | M/N |
| *p16* |  |  |  |  |  |  |  |  |  |  |  |  |  |  |
|  | Ahrendt 1999 | USA | Caucasians | NA | MSP | NA | NA | 1-3 | 50 (24) | NA | 5/23 | 7/19 |  |  |
|  | Kersting 2000 | Germany | Caucasians | 63 | MSP | NA | 39 | 1-4 | 51 (21.6) | 25 (12) |  |  |  |  |
|  | He 2002 | China | Asians | NA | MSP | NA | NA | NA | 21 (33.3) | 18 (0) |  |  |  |  |
|  | Topaloglu 2004 | USA | Caucasians | 50 | MSP | NA | NA | 1-4 | 31 (3.2) | 10 (0) |  |  |  |  |
|  | Kim 2004 | Korea | Asians | 63 | MSP | Yes | 57 | 1-2 | 85 (28.2) | 127 (6.3) |  |  |  |  |
|  | Yang 2004 | China | Asians | NA | MSP | NA | NA | NA | 32 (28.1) | 24 (0) |  |  |  |  |
|  | Grote 2005 | Germany | Caucasians | 64 | QMSP | 71 | 55 | 1-4 | 75 (24) | 64 (0) | 14/25 | 3/25 | 17/50 | 1/25 |
|  | Fraipont 2005 | France | Caucasians | NA | MSP | Yes | NA | 1-2 | 23 (17.4) | 75 (14.7) |  |  |  |  |
|  | Schmiemann 2005 | Germany | Caucasians | 64 | QMSP | 58 | 58 | 1-4 | 85 (11.8) | 102 (0) | 6/16 | 2/33 | 9/67 | 1/18 |
|  | Georgiou 2007 | Greece | Caucasians | 55 | MSP | Yes | NA | NA | 62 (74.2) | 8 (37.5) |  |  | 45/56 | 1/6 |
|  | Hu 2009 | China | Asians | 67 | MSP | NA | NA | 1-4 | 42 (59.5) | 25 (0) |  |  |  |  |
|  | Nikolaidis 2012 | UK | Caucasians | NA | QMSP | 321 | 194 | 1-4 | 333 (16.2) | 322 (0.3) |  |  |  |  |
| *RASSF1A* |  |  |  |  |  |  |  |  |  |  |  |  |  |  |
|  | Topaloglu 2004 | USA | Caucasians | 50 | MSP | NA | NA | 1-4 | 31 (12.9) | 10 (0) |  |  |  |  |
|  | Kim 2004 | Korea | Asians | 63 | MSP | Yes | 57 | 1-2 | 85 (37.6) | 127 (3.9) |  |  |  |  |
|  | Schmiemann 2005 | Germany | Caucasians | 64 | QMSP | 58 | 58 | 1-4 | 85 (41.2) | 102 (0) | 4/15 | 11/33 | 20/67 | 15/18 |
|  | Grote 2006 | Germany | Caucasians | NA | QMSP | 125 | NA | 1-4 | 157 (45.9) | 46 (0) | 13/48 | 11/42 | 31/111 | 41/46 |
|  | Yu 2007 | China | Asians | 55 | MSP | NA | 28 | 1-3 | 45 (42.2) | 45 (0) |  |  |  |  |
|  | van der Drift 2012 | The Netherlands | Caucasians | 65 | QMSP | NA | 90 | NA | 129 (42.6) | 28 (0) |  |  |  |  |
|  | Nikolaidis 2012 | UK | Caucasians | NA | QMSP | 321 | 194 | 1-4 | 333 (43.8) | 322 (2.2) |  |  |  |  |
|  | Ilse 2014 | Germany | Caucasians | NA | QMSP | NA | NA | NA | 75 (29.3) | 43 (0) |  |  |  |  |
|  | Ma 2016 | China | Asians | NA | QDs-based FRET | NA | NA | 1-2 | 40 (60) | 10 (10) | 10/16 | 14/24 |  |  |
| *APC* |  |  |  |  |  |  |  |  |  |  |  |  |  |  |
|  | Topaloglu 2004 | USA | Caucasians | 50 | MSP | NA | NA | 1-4 | 31 (16.1) | 10 (0) |  |  |  |  |
|  | Grote 2004 | Germany | Caucasians | NA | QMSP | NA | NA | 1-4 | 155 (29) | 67 (1.5) | 12/47 | 14/41 | 38/109 | 7/46 |
|  | Fraipont 2005 | France | Caucasians | NA | MSP | Yes | NA | 1-2 | 14 (7.1) | 51 (7.8) |  |  |  |  |
|  | Schmiemann 2005 | Germany | Caucasians | 64 | QMSP | 58 | 58 | 1-4 | 85 (16.5) | 102 (1) | 3/16 | 8/33 | 14/67 | 0/18 |
|  | Ilse 2014 | Germany | Caucasians | NA | QMSP | NA | NA | NA | 75 (13.3) | 43 (0) |  |  |  |  |
| *SHOX2* |  |  |  |  |  |  |  |  |  |  |  |  |  |  |
|  | Schmidt 2010 | Germany | Caucasians | 67 | RTPCR | 260 | NA | 1-4 | 281 (67.6) | 242 (4.9) | 84/103 | 51/109 | 161/249 | 28/29 |
|  | Dietrich 2012 | Germany | Caucasians | NA | RTPCR | Yes | NA | NA | 100 (78) | 104 (3.8) | 21/23 | 17/22 | 45/55 | 28/30 |
|  | Ilse 2014 | Germany | Caucasians | NA | QMSP | NA | NA | NA | 75 (64) | 43 (2.3) | 13/16 | 17/26 | 36/54 | 9/11 |
|  | Konecny 2016 | Slovakia | Caucasians | NA | RTPCR | Yes | NA | 1-4 | 37 (89.2) | 26 (15.4) |  |  |  |  |
| *RARB2* |  |  |  |  |  |  |  |  |  |  |  |  |  |  |
|  | Topaloglu 2004 | USA | Caucasians | 50 | MSP | NA | NA | 1-4 | 31 (0) | 10 (0) |  |  |  |  |
|  | Grote 2005 | Germany | Caucasians | 64 | QMSP | 71 | 55 | 1-4 | 75 (56) | 64 (12.5) | 13/25 | 14/25 | 27/50 | 15/25 |
|  | Schmiemann 2005 | Germany | Caucasians | 64 | QMSP | 58 | 58 | 1-4 | 83 (48.2) | 102 (20.6) | 8/15 | 11/33 | 28/66 | 12/17 |
| *FHIT* |  |  |  |  |  |  |  |  |  |  |  |  |  |  |
|  | Kim 2004 | Korea | Asians | 63 | MSP | Yes | 57 | 1-2 | 85 (32.9) | 127 (28.3) |  |  |  |  |
|  | · | France | Caucasians | NA | MSP | Yes | NA | 1-2 | 22 (31.8) | 74 (28.4) |  |  |  |  |
| *MGMT* |  |  |  |  |  |  |  |  |  |  |  |  |  |  |
|  | Topaloglu 2004 | USA | Caucasians | 50 | MSP | NA | NA | 1-4 | 31 (22.6) | 10 (0) |  |  |  |  |
|  | Fraipont 2005 | France | Caucasians | NA | MSP | Yes | NA | 1-2 | 24 (12.5) | 78 (2.6) |  |  |  |  |
| *RARB* |  |  |  |  |  |  |  |  |  |  |  |  |  |  |
|  | Kim 2004 | Korea | Asians | 63 | MSP | Yes | 57 | 1-2 | 85 (36.5) | 127 (12.6) |  |  |  |  |
|  | Nikolaidis 2012 | UK | Caucasians | NA | QMSP | 321 | 194 | 1-4 | 333 (28.5) | 322 (8.7) |  |  |  |  |
| *CDH13* |  |  |  |  |  |  |  |  |  |  |  |  |  |  |
|  | Kim 2004 | Korea | Asians | 63 | MSP | Yes | 57 | 1-2 | 85 (34.1) | 127 (3.1) |  |  |  |  |
|  | Nikolaidis 2012 | UK | Caucasians | 69 | QMSP | 186 | 114 | 1-4 | 194 (15.5) | 213 (20.2) |  |  |  |  |
| *DAPK* |  |  |  |  |  |  |  |  |  |  |  |  |  |  |
|  | Fraipont 2005 | France | Caucasians | NA | MSP | Yes |  | 1-2 | 24 (12.5) | 77 (14.3) |  |  |  |  |
|  | Nikolaidis 2012 | UK | Caucasians | 69 | QMSP | 186 | 114 | 1-4 | 194 (5.7) | 213 (2.8) |  |  |  |  |
| *GSTP1* |  |  |  |  |  |  |  |  |  |  |  |  |  |  |
|  | Topaloglu 2004 | USA | Caucasians | 50 | MSP | NA | NA | 1-4 | 31 (3.2) | 10 (0) |  |  |  |  |
| *CDH1* |  |  |  |  |  |  |  |  |  |  |  |  |  |  |
|  | Topaloglu 2004 | USA | Caucasians | 50 | MSP | NA | NA | 1-4 | 31 (41.9) | 10 (0) |  |  |  |  |
| *SEMA3B* |  |  |  |  |  |  |  |  |  |  |  |  |  |  |
|  | Grote 2005 | Germany | Caucasians | 64 | QMSP | 71 | 55 | 1-4 | 75 (88) | 25 (92) | 24/25 | 21/25 | 45/50 | 21/25 |
| *TERT* |  |  |  |  |  |  |  |  |  |  |  |  |  |  |
|  | Nikolaidis 2012 | UK | Caucasians | NA | QMSP | 321 | 194 | 1-4 | 333 (61.6) | 322 (11.5) |  |  |  |  |
| *WT1* |  |  |  |  |  |  |  |  |  |  |  |  |  |  |
|  | Nikolaidis 2012 | UK | Caucasians | NA | QMSP | 321 | 194 | 1-4 | 333 (42.9) | 322 (5.6) |  |  |  |  |
| *CYGB* |  |  |  |  |  |  |  |  |  |  |  |  |  |  |
|  | Nikolaidis 2012 | UK | Caucasians | NA | QMSP | 321 | 194 | 1-4 | 333 (15.3) | 322 (5) |  |  |  |  |
| *p73* |  |  |  |  |  |  |  |  |  |  |  |  |  |  |
|  | Nikolaidis 2012 | UK | Caucasians | 69 | QMSP | 186 | 114 | 1-4 | 194 (15.5) | 213 (8) |  |  |  |  |
| *TMEFF* |  |  |  |  |  |  |  |  |  |  |  |  |  |  |
|  | Nikolaidis 2012 | UK | Caucasians | 69 | QMSP | 186 | 114 | 1-4 | 194 (7.2) | 213 (6.6) |  |  |  |  |
| *HOXA9* |  |  |  |  |  |  |  |  |  |  |  |  |  |  |
|  | Ma 2016 | China | Asians | NA | QDs-based FRET | NA | NA | 1-2 | 40 (70) | 10 (0) | 12/16 | 16/24 |  |  |
| *PCDHGB6* |  |  |  |  |  |  |  |  |  |  |  |  |  |  |
|  | Ma 2016 | China | Asians | NA | QDs-based FRET | NA | NA | 1-2 | 40 (57.5) | 10 (10) | 8/16 | 15/24 |  |  |

NA: not applicable; N: sample size; M: methylation; MSP: methylation-specific polymerase chain reaction; QMSP: quantitative methylation-specific polymerase chain reaction; QDs-based FRET: the developed quantum dots-based (QDs-based) fluorescence resonance energy transfer (FRET) nanosensor technique; RT-PCR: the real-time PCR based HeavyMethyl technology; SCC: squamous cell carcinoma; AC: adenocarcinoma; NSCLC: small cell lung cancer; SCLC: small cell lung cancer.
